# Computational optimization of food formulation and extrusion conditions for nutritional quality and antioxidant capacity

**DOI:** 10.1016/j.crfs.2026.101509

**Published:** 2026-07-22

**Authors:** Xu Zhou, Keer Ni, Pranav Gupta, Ilias Tagkopoulos

**Affiliations:** a Department of Computer Science & Genome Center, University of California, Davis, 95616, USA; b USDA/NSF AI Institute for Next Generation Food Systems (AIFS), Davis, 95616, USA

**Keywords:** AI, Extrusion, Formulation, Nutrient Rich Foods Index, Bioactivity, Mechanistic model, Machine learning, Optimization

## Abstract

Processed foods are important to modern dietary patterns, yet current classification systems are qualitative and provide limited guidance for designing healthier products. Here, we present a computational framework integrating an ingredient-level nutrient and bioactive compound database, mechanistic kinetic models, a machine learning predictor, and global optimization to improve nutritional quality and antioxidant capacity of processed foods, using extrusion as a case study. An large language model (LLM)-assisted data extraction workflow curated a database of 503 ingredient records from 2000 peer-reviewed papers and commercial supplier data. Bioactive and nutrient concentrations varied by several orders of magnitude among ingredients, with a median within-ingredient coefficient of variation of 36% across supplier-level entries. Ingredient selection alone accounted for a mean Nutrient Rich Foods index (NRF_9_._3_) variability of 28% across formulations. Under a simulated reference extrusion condition, the model predicted that NRF_9_._3_ decreased while Ferric Reducing Antioxidant Power (FRAP) increased across all 20 formulations. Variability in ingredient sourcing typically contributed 3 to 11times more to changes in NRF_9_._3_ than extrusion, although extrusion had a greater effect in formulations rich in heat-labile vitamins. Formulation-specific optimization of extrusion temperature and residence time was predicted to improve the combined NRF_9_._3_–FRAP score by a mean of 10% (up to 44%) over the reference condition. As a whole, this work provides a generalizable framework that integrates formulations and process parameters for systematic optimization of nutritional and bioactivity targets in food processing.

## Highlights

• A computational framework links formulation, extrusion, nutrition and health.
• A curated database integrates nutrient and phytochemical data.
• Ingredient sourcing variability often exceeded processing effects on nutrition.
• Extrusion lowers nutritional quality but increases antioxidant capacity.
• Process optimization reduced nutrition loss while enhancing antioxidant capacity.

## Graphical abstract

**Figure:**
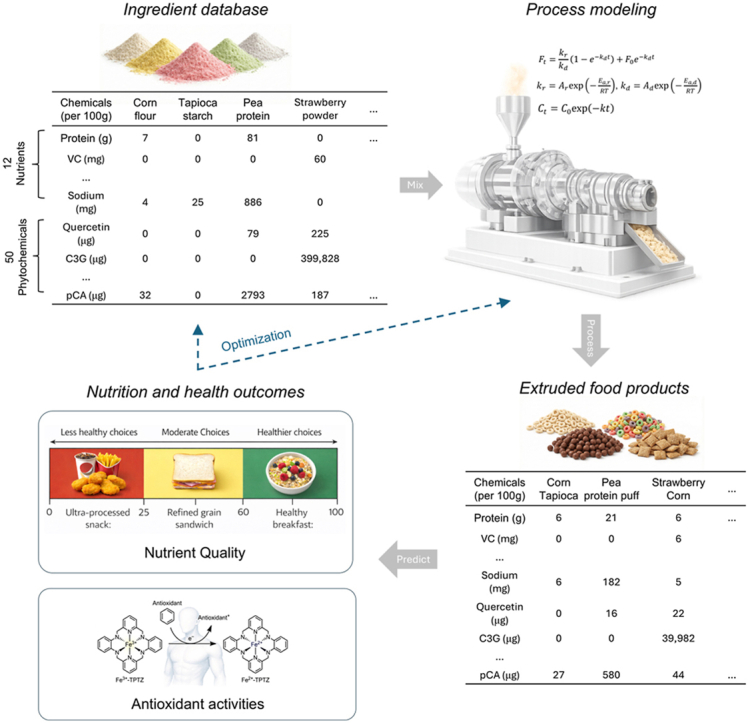

## 1 Introduction

Processed foods are a major component of modern diets, accounting for more than half of daily caloric intake in the United States (Juul et al., 2022; Chen et al., 2023). Meanwhile, diet-associated chronic diseases such as obesity, type 2 diabetes, and cardiovascular disease are increasingly prevalent (Thompson, 2021). The trends have intensified interest in understanding how processed foods influence human nutrition and health. However, many current frameworks for evaluating processed foods are qualitative and do not clearly link specific ingredients or manufacturing to nutritional quality or physiological responses. One widely used framework is the NOVA (Monteiro et al., 2018) classification system, which groups foods into four categories, ranging from unprocessed or minimally processed foods to ultra-processed foods. Yet NOVA can conflate formulation choices with processing-induced transformations, providing limited guidance for processing healthier foods (Ahrné et al., 2025; Raita et al., 2025).

Extensive experimental studies have examined how formulation and processing conditions influence food quality and nutrition (Nayak et al., 2015; Camire et al., 1990; Kharat et al., 2019; Kowalski et al., 2016; Singh et al., 2007). Among high-throughput food manufacturing methods, extrusion is widely used to produce ready-to-eat cereals, snacks, and plant-based products. In extrusion processing, heat and mechanical shear are applied simultaneously, driving multiple transformations such as starch gelatinization, protein denaturation, and product expansion (Alam et al., 2016). Several studies provide useful information on how formulation and processing conditions influence starch functionality, protein digestibility, vitamin stability, and nutrient composition (Camire et al., 1990; Singh et al., 2007; Song and Tang, 2023). But most of the results are ingredient-specific, equipment-dependent, and difficult to generalize across formulations and processing conditions. Extrusion can simultaneously degrade heat-sensitive nutrients and modify the food matrix in ways that may alter the release and accessibility of bioactive compounds (Nayak et al., 2015; Song and Tang, 2023; Camire et al., 2007; Song et al., 2022). Product development for nutritious and healthy foods is still empirical and relies on time-consuming and costly trial-and-error.

Thus, we present a predictive framework connecting 1) a curated database of nutrients and bioactive compounds for cereal-relevant ingredients, 2) mechanistic kinetic models that predict processing-induced changes in selected nutrients and bioactive compounds during extrusion, 3) a machine learning model that maps chemical compositions to antioxidant capacity of processed foods, and 4) an optimization of processing conditions to improve nutrition and antioxidant capacity.

## 2 Methods

### 2.1 Food database of nutrients and phytochemicals

We developed a structured database capturing nutritional and bioactive compositions of ingredients. This study focused on cereal extrusion, so we selected the following related ingredients: cereal grains, legume- and plant-based proteins, vegetable and fruit powders, fibers, and lipids (Camire et al., 2007; Song et al., 2022; Perdon et al., 2020; Fulgoni et al., 2009).

#### 2.1.1 Data sources

We used Nutrient-Rich Foods index (NRF_9.3_) (Drewnowski, 2010; Drewnowski and Fulgoni, 2008; Fulgoni et al., 2009) as a nutrition quality metric. The NRF_9.3_ uses nine nutrients to encourage (protein, dietary fiber, vitamins A, C, and E, calcium, iron, potassium, and magnesium) and three nutrients to limit (saturated fat, added sugar, and sodium). NRF_9.3_ can be calculated as follows (Drewnowski and Fulgoni, 2008):

(1) $$\text{NRF}9.3=\sum_{i=1}^{9}\left(\frac{N_{i}}{DV_{i}}\times 100\right)-\sum_{j=1}^{3}\left(\frac{L_{j}}{DV_{j}}\times 100\right)$$

where $N_{i}$ is the amount of nutrient *i* to encourage per 100 kcal, $L_{j}$ is the amount of nutrient *j* to limit per 100 kcal, and $DV_{i},DV_{j}$ are daily value for nutrient *i* or *j* respectively. We collected 12 NRF_9.3_ nutrient data from USDA FDC, TraceGains (https://tracegains.com/) and FoodAtlas (Youn et al., 2024). All entries were standardized to common units (e.g., per 100 g), and ingredient records were harmonized across sources using consistent naming and mapping rules.

We also collected bioactive compound data, particularly antioxidant compounds from peer-reviewed publications. Papers were identified through structured searches in Google Scholar using ingredient-specific and compound-class keywords (for example: “ingredient name” AND “phenolic”). For each ingredient, we screened results in descending relevance until no new eligible quantitative measurements were identified, with a maximum of 100 candidate papers per ingredient. We collected 2,000 papers total. The 12 NRF_9.3_ nutrients were thus obtained at supplier and database level (USDA FoodData Central and TraceGains), whereas bioactive and phenolic concentrations were literature-derived (aggregated across peer-reviewed studies).

#### 2.1.2 Automated workflow for data extraction

Manual extraction is very time-consuming, so we developed an automated workflow for data extraction. The workflow was implemented in n8n (https://n8n.io/), enabling modular orchestration of document ingestion, text extraction, large language model (LLM)–based information extraction, and structured data storage (Dagdelen et al., 2024). For each paper, an LLM (OpenAI GPT- 5.2) identified and normalized data (e.g., composition, concentration values, and metadata) into a predefined schema. Detailed extraction schema can be found in Supplementary Table S2. Post-processing logic was applied to resolve missing, zero, or ambiguous values, ensuring consistency with database constraints. Extracted records were automatically stored by updating structured data repositories, including cloud-based spreadsheets and database files. To assess extraction quality, we manually audited a random 10% sample of source PDFs (n ∼ 200). Audit items were compared against the normalized database record after harmonization and unit conversion. Agreement was defined as relative error ≤1% or absolute error ≤0.001 in final database units, whichever was larger. Of 609 extracted records, 106 (17.4%) were removed during manual review due to incomplete nutrient coverage (≥60% missing fields across required nutrients), yielding a final curated database of 503 records.

### 2.2 Formulation

We built 20 dry-mix formulations from the 28 ingredients in our database (Supplementary Table S1). Ingredient ranges were constrained by extrusion and breakfast cereal literature to ensure feasibility and relevance. We then used an LLM (OpenAI GPT-5.2) to propose diverse candidate recipes within these bounds and feasibility rules (e.g., sum-to-one and conservative limits for lipid- and fiber-rich ingredients). The generation prompt was provided in Supplementary. Redundant recipes were removed and the final set was manually curated for broad coverage.

### 2.3 Mechanistic modeling of extrusion

#### 2.3.1 Kinetic models for nutrients

NRF_9.3_ includes 12 nutrients. We classified these nutrients based on their known thermal stability and dominant transformation mechanisms under extrusion conditions (van Boekel, 2008). Macronutrients and minerals, including protein, dietary fiber, calcium, iron, potassium, magnesium, saturated fat, and sodium are generally stable under common extrusion conditions; therefore, we assumed their amounts were quantitatively retained during extrusion. Quantitative retention refers to total composition only; extrusion may still alter protein digestibility, the bioaccessibility of nutrients and bioactive compounds, and the ratio of soluble to insoluble dietary fiber, which NRF_9.3_ score does not capture. Heat-sensitive vitamins (vitamins A, C, and E) were modeled using first-order degradation kinetics and Arrhenius equation (van Boekel, 2008):

(2) $$k=A\;\exp\left(-\frac{E_{a}}{RT}\right)$$

where k is the reaction rate constant (${\text{s}}^{-1}$), A is the pre-exponential factor (${\text{s}}^{-1}$), $E_{a}$ is the activation energy (${\text{J}\;\text{mol}}^{-1}$), R is the universal gas constant, and T is the absolute temperature (K). The concentration of a nutrient after extrusion time *t* was:

(3) $$C_{t}=C_{0}\;\exp\left(-kt\right)$$

where $C_{0}$ and $C_{t}$ represent initial and post-processing concentrations, respectively. Kinetic parameters (activation energies and pre-exponential factors) for vitamins A, C, and E were sourced from published studies (Riaz et al., 2009; Coelho, 2002) (Table S3), and applied uniformly across all 28 ingredients in the absence of ingredient-specific calibration data. Although vitamin degradation depends on the ingredient matrix, moisture content, pH, oxygen exposure, and other processing factors, this simplified approach was used as an initial model because condition- and ingredient-specific data were limited. Future work can improve the model by incorporating experimentally calibrated kinetic parameters for individual ingredients and conditions.

#### 2.3.2 Kinetic models for bioactives

Bioactive transformations during extrusion were modeled with a focus on phenolic acids, the dominant bioactive compounds in cereal grains and a major contributor to antioxidant capacity (Nayak et al., 2015; Song and Tang, 2023; Song et al., 2022; Zeng et al., 2016). Phenolic acids occur in both bound (covalently linked to cell wall polysaccharides and proteins) and free forms (Song et al., 2022). Because commonly used in vitro antioxidant assays (e.g., DPPH, ABTS, FRAP) quantify only solvent-extractable compounds, the free phenolic fraction was treated as the functionally relevant bioactive pool for health-related outcome prediction (Benzie and Strain, 1996; Huang et al., 2005). Experimental studies indicate that extrusion induces two competing processes affecting free phenolic acids: (i) matrix disruption–driven release of bound phenolics into the free form, and (ii) thermal degradation of free phenolics at elevated temperatures (Nayak et al., 2015; Song and Tang, 2023; Song et al., 2022; Zeng et al., 2016). Bound phenolics were modeled as a finite reservoir released at a zero-order rate, while free phenolics were simultaneously generated by release and consumed by first-order degradation:

(4) $$\frac{dC_{b}}{dt}=-k_{b}\left(T\right)$$

(5) $$\frac{dC_{f}}{dt}=k_{b}\left(T\right)-k_{f}\left(T\right)C_{f}$$

where *C*_*b*_ and *C*_*f*_ were bound and free phenolic concentrations (${\text{mg}\;\text{kg}}^{-1}$), respectively, *k*_*b*_ was the release rate constant (${\text{mg}\;\text{kg}}^{-1}$ ${\text{s}}^{-1}$), and *k*_*f*_ was the degradation rate constant (${\text{s}}^{-1}$). Both rate constants were temperature-dependent and described by Arrhenius kinetics (Eq. (2)). Initial conditions were $C_{b}\left(0\right)=C_{b,0}$ and $C_{f}\left(0\right)=C_{f,0}$.

Because the bound pool is finite, release ceases once *C*_*b*_ is depleted. The depletion time is

(6) $$t^{*}=\frac{C_{b,0}}{k_{b}\left(T\right)}$$

Then the predicted exit free concentration is:

(7) $$C_{f,\text{out}\;}=\left\{ \begin{array}{c} \left(C_{f,0}-\frac{k_{b}}{k_{f}}\right)e^{-k_{f}t_{R}}+\frac{k_{b}}{k_{f}},0\leq t_{R}\leq t^{*}\\ \left[\left(C_{f,0}-\frac{k_{b}}{k_{f}}\right)e^{-k_{f}t^{*}}+\frac{k_{b}}{k_{f}}\right]e^{-k_{f}\left(t_{R}-t^{*}\right)},t_{R}>t^{*} \end{array}\right.$$

Complete derivation can be found in the Supplementary Material. Phenolic kinetic parameters were similarly sourced from published extrusion studies and applied uniformly across ingredients. This approach captures the dominant thermal release and degradation mechanisms but does not account for ingredient-specific matrix effects, ingredient–ingredient interactions, moisture content variation, or shear-driven transformations; predictions therefore reflect aggregate behavior within each ingredient class rather than precise compound-level outcomes for specific raw material lots.

### 2.4 Antioxidant capacity modeling

#### 2.4.1 Antioxidant capacity dataset

To predict formulation-level antioxidant capacity, we constructed a paired dataset linking food chemical profiles to in vitro antioxidant measurements, termed the Bioactivity Food Link (BFL). Chemical composition features were assembled from our curated composition database (FoodAtlas (Youn et al., 2024), and literature-derived bioactive profiles), and assay endpoints were compiled from peer-reviewed studies reporting antioxidant capacity for foods and extrusion-relevant ingredients under defined processing conditions. The BFL dataset included n = 275 food samples with one or more of the following assays: FRAP, DPPH, ABTS, and ORAC. For each BFL sample, we therefore generated processing-adjusted chemical features by propagating initial composition through the kinetic models. The resulting post-processing composition vector served as the model input for predicting antioxidant capacity.

#### 2.4.2 Machine learning model

We trained a Bioactivity Prediction Model (BPM) to predict antioxidant capacity from the processing-adjusted chemical feature vector. BPM was implemented as a Random Forest (Breiman, 2001), selected for its ability to capture nonlinear relationships and interactions among compositional features without requiring parametric assumptions. The primary endpoint reported in this work is FRAP, while DPPH, ABTS, and ORAC were used as complementary endpoints where available.

#### 2.4.3 Model evaluation

Model generalization was assessed using 100 iterations of repeated random subsampling (80/20 train/test splits at the formulation level). Hyperparameters were optimized by grid search within each training fold. The overall prediction framework is illustrated in Supplementary Fig. S1. Across held-out test sets, the model achieved R^2^ = 0.75, PCC = 0.87, and RMSE = 0.42 on log-transformed FRAP values (Supplementary Fig. S2). Feature importance was assessed using mean decrease in impurity averaged across bootstrap runs, and features with consistently below-median importance were excluded (details in Supplementary Methods). Of the 275 food samples in the BFL dataset, only 30 corresponded to extrusion-specific conditions; the remaining samples derived from non-extruded or broadly processed foods. When evaluated specifically on these 30 extrusion-specific samples using a leave-one-study-out scheme, the model explained approximately 46% of the variance (R^2^ = 0.46; Supplementary Table S4). Model generalization to novel extrusion-specific compositions therefore carries additional uncertainty beyond the bootstrap estimate, and predictions should be interpreted as indicative rather than quantitatively precise until validated against prospective extrusion experiments. A comparison of ten regression algorithms confirmed that Random Forest achieved the highest aggregate performance across all evaluation metrics (Supplementary Fig. S3).

### 2.5 Model-based optimization of calculated NRF9.3 and predicted antioxidant capacity

For each fixed formulation, we optimized two model variables, namely extrusion temperature and mean residence time, to maximize a weighted objective combining calculated NRF9.3 and predicted FRAP. For formulation k (composition $x_{k}$ from Supplementary Table S1), the decision variables were extrusion temperature T and mean residence time t. For any candidate (T,t), we used kinetic and machine learning models to compute $NRF9.3\left(x_{k},T,t\right)$ and $FRAP\left(x_{k},T,t\right)$. We defined a weighted objective that combines min–max normalized metrics over the feasible (T,t) search space:

(8) $$J_{k}\left(T,t\right)=\lambda \hat{{NRF}_{9.3}}\left(x_{k},T,t\right)+\left(1-\lambda \right)\hat{FRAP}\left(x_{k},T,t\right)$$

where λ∈[0,1] was the trade-off between nutrient retention and antioxidant capacity, and $\overbrace{}$ was min–max normalization; we used λ=0.5 as a demonstration. The optimal processing condition for each formulation was $\left(T_{k}^{*},t_{k}^{*}\right)=\arg\max_{T,t}J_{k}\left(T,t\right)$;

Because $J_{k}\left(T,t\right)$ is nonlinear and may be multimodal, we used global optimization methods. Differential Evolution (Storn and Price, 1997) and dual annealing (Simulated Annealing) were used as primary solvers because they do not require gradients and are less sensitive to initialization. Grid search was used for visualization and coarse benchmarking, and local solvers were used diagnostically to assess sensitivity to initialization (Madoumier et al., 2019). Robustness was checked by repeating global optimization with different random seeds and verifying solutions by local refinement and neighborhood sensitivity analysis. Although the problem involves only two decision variables, we retained global solvers for generality and reproducibility across formulations; convergence was confirmed by exhaustive grid search, multiple random seeds, and local refinement, which agreed on the same optima. The optimization outputs were formulation-specific optimal extrusion settings $\left(T_{k}^{*},t_{k}^{*}\right)$ and the corresponding predicted NRF_9.3_ and FRAP values.

### 2.6 Uncertainty propagation analysis

To quantify how ingredient sourcing variability propagates to formulation-level nutritional quality, we performed a Monte Carlo simulation with 5000 iterations per formulation. For each iteration, the concentration of each NRF_9_._3_ nutrient in each ingredient was sampled from a normal distribution parameterized by the mean and standard deviation reported in the database (Supplementary Table S2). Where supplier-level standard deviations were unavailable, a conservative coefficient of variation of 10% was applied. Sampled concentrations were constrained to be non-negative. For each draw, formulation-level nutrient concentrations were computed as the weighted sum over ingredient fractions, and NRF_9_._3_ was calculated per Eq. (1). The same procedure was repeated after applying the kinetic degradation model (Eqs. (2) and (3)) at the reference extrusion condition (140 °C, 30 s) to obtain post-processing NRF_9_._3_ distributions. To assess the relative importance of ingredient sourcing versus processing, we computed the ratio of the sourcing-driven standard deviation (σ_sourcing, from the raw NRF_9_._3_ distribution) to the absolute mean processing-induced shift (|Δ_processing|, the difference between raw and processed NRF_9_._3_ means). Per-nutrient contributions to NRF_9_._3_ uncertainty were assessed by computing the coefficient of variation of each nutrient's NRF_9_._3_ component across the 5000 draws, averaged over all 20 formulations. Because the NRF calculation involves division by energy and nutrient concentrations are sampled from truncated normal distributions, the Monte Carlo mean NRF can differ slightly from the deterministic value in Table 1; however, this discrepancy does not affect the sourcing-to-processing ratio or per-nutrient CV decomposition reported here. A sensitivity analysis confirmed robustness to the default CV assumption: varying it from 5% to 40% left all eight sourcing-dominated formulations unchanged and shifted the median sourcing-to-processing ratio only modestly, from 3.3 to 4.2 (Supplementary Fig. S4).

**Table 1:** **Nutritional quality and antioxidant capacity before and after extrusion, and under optimized processing conditions across 20 formulations**. For each formulation (ID 1–20), the table reports the calculated Nutrient Rich Food (NRF) score and predicted Ferric Reducing Antioxidant Power before processing, after a fixed baseline extrusion condition (140 °C, 30 s), and after formulation-specific optimized extrusion settings (temperature, T; time, t).

	Formulation	Raw			After baseline processing			After optimized processing (Temperature and Time)				
		NRF_9.3_	FRAP	Overall score (J^b^)	NRF_9.3_	FRAP a	J^b^	T(°C)	t(s)	NRF_9.3_	FRAP a	J^b^
1	Corn-Tapioca Standard	12.90	0.32	0.16	12.67	0.43	0.30	109	34	12.68	0.47	0.34
2	Gluten-Free Rice Crisp	21.13	0.42	0.36	20.93	0.71	0.55	138	24	20.97	0.75	0.56
3	Multi-Grain Textural Hybrid	26.32	0.40	0.38	25.85	0.48	0.46	144	40	26.16	0.48	0.47
4	Low-Density Carrier	14.57	0.37	0.25	14.33	0.44	0.33	109	31	14.39	0.49	0.37
5	Pea Protein Isolate Puff	26.62	0.36	0.33	26.37	0.44	0.43	103	21	26.46	0.49	0.47
6	Chickpea Legume Blend	26.47	0.43	0.43	25.82	0.55	0.51	156	40	25.98	0.78	0.61
7	Mung Bean Crisp	37.20	0.28	0.32	26.10	0.33	0.34	108	22	32.90	0.33	0.39
8	Peanut Flour Blend	20.87	0.36	0.30	20.69	0.44	0.38	106	26	20.74	0.48	0.42
9	Sorghum-Millet Drought Blend	72.20	0.42	0.80	71.78	0.47	0.84	132	38	72.06	0.47	0.84
10	Buckwheat Matrix	23.24	0.33	0.27	22.90	0.44	0.40	113	22	23.13	0.48	0.44
11	Rye-Oat Fiber Crunch	35.63	0.40	0.46	34.77	0.44	0.50	109	22	35.30	0.49	0.55
12	Pea Hull Fiber Fortified	15.63	0.36	0.25	15.31	0.48	0.37	130	35	15.52	0.52	0.40
13	Strawberry-Corn Red Puff	46.09	0.27	0.32	19.79	0.33	0.23	117	21	26.36	0.33	0.30
14	Apple-Oat Ring	28.86	0.39	0.40	26.43	0.45	0.44	106	57	27.97	0.46	0.47
15	Almond-Rice Marzipan	22.97	0.40	0.36	19.70	0.72	0.54	105	22	20.37	0.74	0.55
16	Barley-Berry Antioxidant	59.41	0.31	0.53	34.18	0.33	0.36	138	27	40.75	0.33	0.41
17	Tomato-Basil Corn Sphere	30.32	0.25	0.15	21.97	0.29	0.17	108	59	24.06	0.30	0.22
18	Garlic-Onion Rice Pillow	21.87	0.29	0.17	20.96	0.34	0.25	120	56	21.54	0.41	0.36
19	Spicy Chickpea Savory Crunch	29.54	0.28	0.21	28.07	0.33	0.31	119	60	28.45	0.34	0.32
20	Garden Multigrain Complex	37.00	0.28	0.28	32.14	0.31	0.31	135	25	33.78	0.32	0.33

a FRAP value units are (-log(mol/100g))^−1^.

b For λ=0.5*,*$J_{k}\left(T,t\right)=\lambda \hat{{NRF}_{9.3}}\left(x_{k},T,t\right)+\left(1-\lambda \right)\hat{FRAP}\left(x_{k},T,t\right)$

## 3 Results

### 3.1 An ingredient-level compositional database of nutrients and phytochemicals

**Fig. 1:**
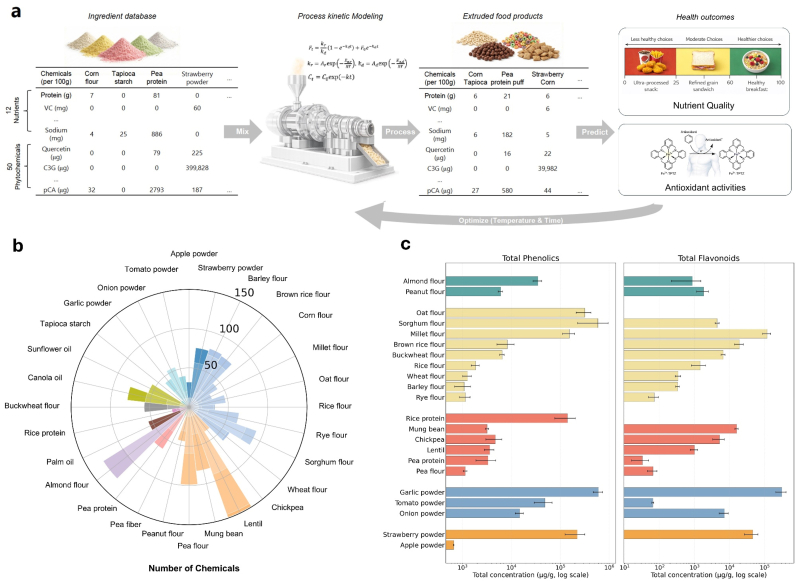
**Computational framework linking ingredient composition, extrusion processing, NRF_9.3_ nutrition and FRAP antioxidant metrics. (a)** Pipeline from ingredient chemical database, process kinetic modeling to nutrient quality and antioxidant activities. **(b)** Number of quantified chemicals available per ingredient in the database. **(c)** Ingredient-level distributions of total flavonoids and total phenolic acids.

We built an ingredient-level compositional database (Supplementary Table S2) by integrating nutrient and phytochemical compound data. Fig. 1b shows that chemical coverage varied across ingredients, ranging from fewer than 20 quantified chemicals in low-complexity ingredients such as pea fiber and tapioca starch to nearly 150 in lentil. This uneven coverage likely reflects not only real compositional differences but also differences in analytical depth in literature.

Total phenolics and total flavonoids varied by several orders of magnitude across ingredients (Fig. 1c). Garlic powder and sorghum flour were among the most phenolic-rich ingredients, with high total phenolics also observed for oat flour, millet flour, rice protein, tomato powder, and strawberry powder (e.g., total phenolics ranged from ∼300 μg/g in lentil to >161,000 μg/g in almond flour; a range spanning more than two orders of magnitude across ingredients). Flavonoid patterns were related but not identical: garlic powder showed the highest total flavonoids, while sorghum flour, millet flour, strawberry powder, onion powder, mung bean, and chickpea were also relatively rich sources. In contrast, apple powder and several lower-complexity cereal ingredients were comparatively low. These class-specific patterns indicate that formulation choices can shift the baseline phytochemical profile of an extruded product by multiple orders of magnitude before processing.

In addition to between-ingredient differences, the database includes supplier-level nutrition data for the ingredients, comprising 391 total entries (Supplementary Fig. S5). Across 219 ingredient–nutrient pairs with three or more supplier entries, the median coefficient of variation (CV) was 36%, and 37% of pairs exceeded a CV of 50%. No ingredient showed consistently low variability across all nutrients. Protein was the most stable nutrient (median CV = 11%), whereas fiber (median CV = 39%) and several minerals showed much larger spreads. Micronutrient variability was particularly high: vitamin C exhibited a median CV of 88%, vitamin E 90%, and vitamin A 175%. Supplier-level nutrient profiles through the 20 formulations showed that ingredient sourcing alone introduced a mean NRF_9_._3_ CV of 28% ± 2% (SEM) across formulations, confirming that raw-material selection is a primary driver of nutritional uncertainty and must be considered in predictive modeling.

### 3.2 NRF_9.3_ and antioxidant capacity are predicted to change in opposite directions under extrusion

**Fig. 2:**
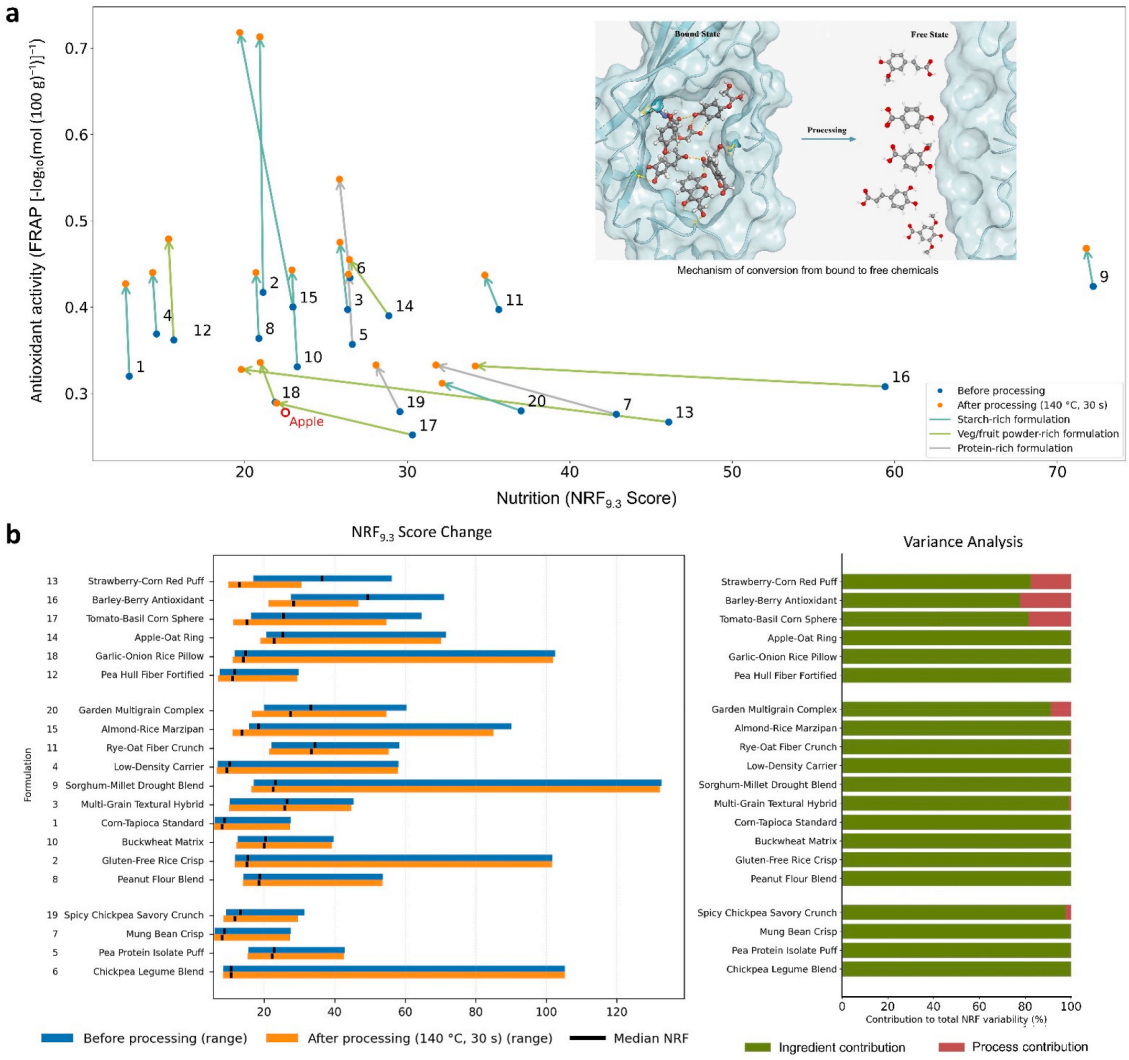
**Predicted effects of extrusion on NRF_9.3_ and FRAP across 20 formulations. (a)** NRF_9.3_ score and FRAP values for each formulation before and after simulated extrusion at 140 °C for 30 s; average ingredient composition values were used as input. **(b)** NRF_9.3_ score before vs. after extrusion propagating ingredient compositional variability (reported ranges from commercial suppliers).

The model predicted that extrusion at the reference condition (140 °C, 30 s) reduced NRF_9_._3_ in the selected 20 formulations (mean −10.7%, range −1% to −57%) while simultaneously increasing predicted FRAP in all 20 formulations (mean + 25%, range +6% to +80%) (Fig. 2a). Fruit- and vegetable-enriched formulations showed substantially greater NRF_9_._3_ losses under extrusion (mean −25.5%) than starch-dominant (mean −1.5%) or protein-dominant systems (mean −7.3%), a markedly larger predicted loss (descriptive comparison across the 20 model-generated formulations). This pattern is consistent with the higher initial concentrations of heat-labile vitamins A, C, and E in these formulations, which are the three limiting nutrients for NRF_9_._3_ reduction under first-order thermal degradation kinetics. These opposite directions follow directly from the model structure (first-order vitamin degradation and release-dominated phenolic kinetics).

**Fig. 3:**
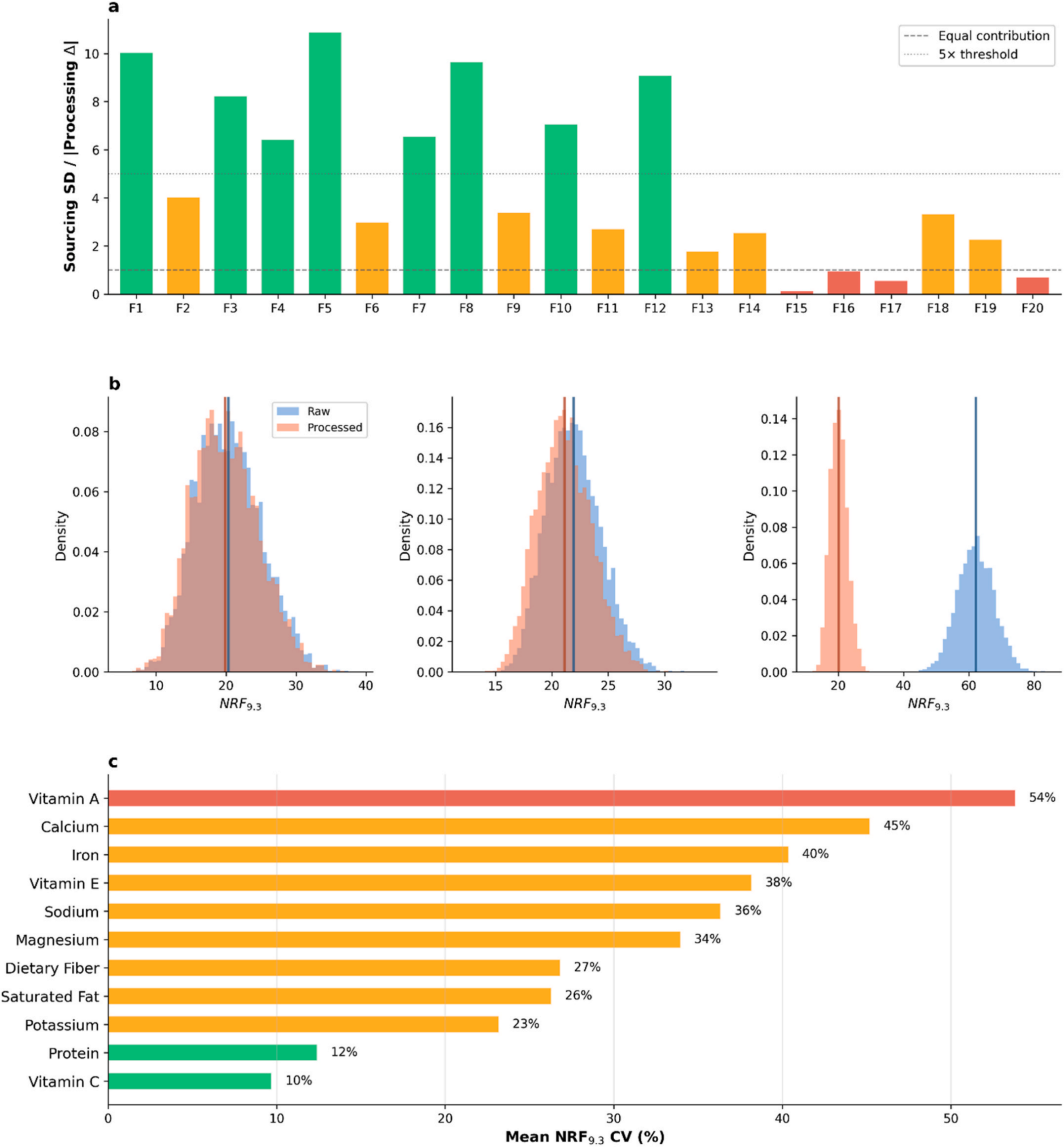
**Ingredient sourcing uncertainty is predicted to dominate processing-induced NRF_9.3_ change in a formulation-dependent manner. (a)** Sourcing-to-processing ratio (σ_sourcing_/|Δ_processing_|) for each formulation. Green: sourcing-dominated (ratio ≥5); orange: mixed (1–5); red: processing-dominated (<1). Dashed lines indicate equal contribution (ratio = 1) and the 5×threshold. **(b)** NRF_9.3_ distributions under ingredient sourcing uncertainty for three representative formulations spanning the sourcing-to-processing spectrum: sourcing-dominated (left), mixed (center), and processing-dominated (right). Blue: unprocessed; orange: extruded at 140 °C, 30 s. Vertical lines indicate distribution means. **(c)** Per-nutrient contribution to NRF_9.3_ uncertainty, measured as the mean coefficient of variation of each nutrient's NRF_9.3_ component across all 20 formulations. All panels are based on Monte Carlo simulation (n = 5000 draws per formulation).

Ingredient sourcing introduced additional variability (Fig. 2b). Supplier-driven NRF_9_._3_ ranges varied widely across formulations, and for most, sourcing-driven variability exceeded the processing-induced shift; variance decomposition showed that ingredient sourcing accounted for ≥85% of total NRF_9_._3_ variability even in the most processing-sensitive formulations, making it at least 5-fold more important than extrusion-induced changes in terms of variance. Monte Carlo uncertainty propagation confirmed and refined this finding (Fig. 3). The sourcing-to-processing ratio, defined as σ_sourcing/|Δ_processing|, varied widely across formulations (Fig. 3a): eight formulations exceeded a ratio of 5 (sourcing-dominated; green), nine fell between 1 and 5 (mixed; orange), and three fell below 1 (processing-dominated; red). Formulations dominated by starch, protein, or fiber (e.g., F#1, F#5, F#8, F#12) showed ratios above 8, indicating that the NRF_9_._3_ uncertainty introduced by ingredient sourcing alone was an order of magnitude larger than the processing-induced shift. In contrast, formulations with high concentrations of heat-labile vitamins (F#15, F#17, F#20) had ratios below 1, meaning that extrusion-driven vitamin degradation exceeded sourcing variability and was the primary determinant of final NRF_9_._3_. The median ratio across all 20 formulations was 3.4; this classification was robust to the assumed default CV for ingredients lacking supplier-level standard deviations (Supplementary Fig. S4). This formulation-dependent pattern is illustrated in Fig. 3b, which shows raw and processed NRF_9_._3_ distributions for three representative formulations: a sourcing-dominated case with near-complete overlap between raw and processed distributions, a mixed case with partial separation, and a processing-dominated case where the processed distribution is shifted well below the raw distribution with minimal overlap. Decomposition of NRF_9_._3_ uncertainty by nutrient (Fig. 3c) revealed that vitamin A contributed the most instability (mean CV = 54% across formulations), followed by calcium (45%), iron (40%), and vitamin E (38%). Protein and vitamin C were the most stable components (CV = 12% and 10%, respectively). This nutrient-level ranking identifies specific sourcing targets: tightening supplier specifications for vitamin A and mineral content would yield the largest reductions in formulation-level nutritional uncertainty. Full NRF_9_._3_ distributions with 95% confidence intervals for all 20 formulations are provided in Supplementary Figs. S6 and S7.

### 3.3 Optimization is predicted to improve the combined nutrition–antioxidant score by up to 44%

**Fig. 4:**
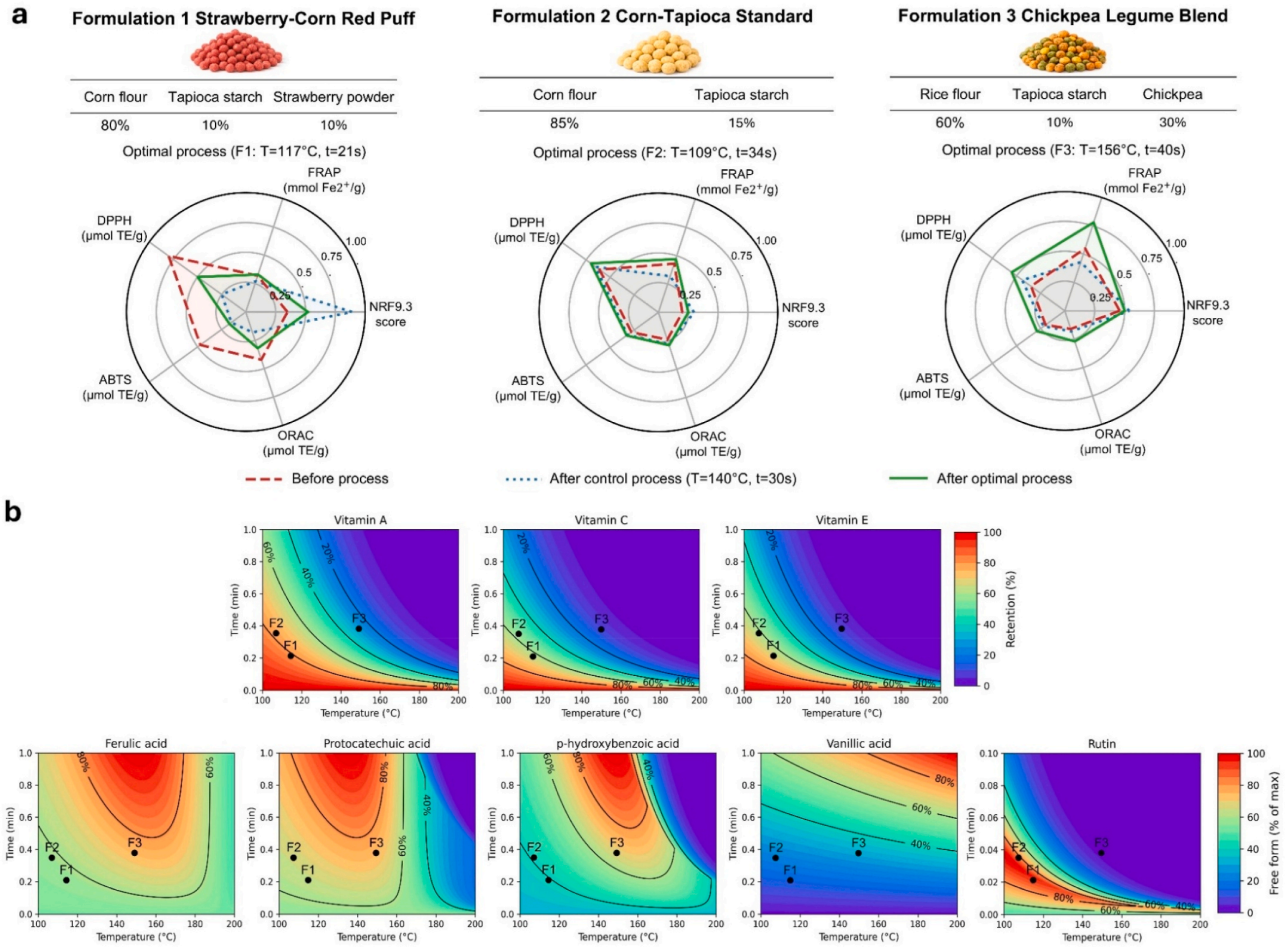
**Optimization and mechanistic insights.** (a) Three extruded product examples with model-identified temperature–residence time optima that maximize the weighted objective combining calculated NRF_9.3_ and predicted FRAP. (b) Kinetics-based predictions of processing-driven changes in selected vitamins and phenolic acids.

Optimization revealed formulation-dependent optimal extrusion conditions spanning 103–156 °C and 21–60 s (Table 1). Optimal settings clustered into three groups: eight formulations favored mild conditions (≤113 °C, ≤35 s), five favored higher temperatures with shorter residence times (117–138 °C, 21–35 s), and seven required more intensive processing at either high temperature or extended residence time. Within the intensive-processing group, two distinct strategies emerged (Fig. 4a): high temperature with moderate residence time (e.g., Chickpea Legume Blend, 156 °C, 40 s) versus lower temperature with extended residence time (e.g., Spicy Chickpea Savory Crunch, 119 °C, 60 s). Formulation-specific optimization was predicted to improve the combined NRF_9_._3_–FRAP score by a mean of 10% (ΔJ = 0.04 ± 0.03) relative to the 140 °C/30 s reference condition. For 18 of 20 formulations, the optimized score was also predicted to exceed the unprocessed value; the two exceptions (Strawberry-Corn Red Puff and Barley-Berry Antioxidant) contain high concentrations of heat-labile vitamins that degrade under any extrusion condition. Because the combined score incorporates predicted FRAP, a value exceeding the unprocessed baseline reflects the model objective under its assumptions and does not by itself indicate a nutritionally or physiologically healthier product. The largest predicted gains occurred in bioactive-rich and vitamin-sensitive formulations (up to 44%), while formulations with low initial phytochemical content showed minimal benefit (ΔJ ≈ 0), suggesting that process optimization is most impactful where ingredient composition creates sensitivity to processing conditions.

## 4 Discussion

Existing food composition databases, such as USDA FoodData Central and FoodAtlas (Youn et al., 2024), provide nutrient data for many foods but generally do not pair those data with ingredient-resolved phytochemical profiles in a way that is directly useful for process design. Our database addresses this gap by combining 12 NRF_9_._3_ nutrients with quantified concentrations for over 600 bioactive compounds across 28 extrusion-relevant ingredients within one standardized framework (Fig. 1a).

The substantial heterogeneity across ingredients means that ingredients contribute different chemical signatures, not simply different total quantities of compounds. This distinction is important because nutrient density and bioactivity represent different dimensions of health. Protein, fiber, vitamins, and minerals contribute to established measures of dietary adequacy, whereas bioactive compounds contribute to functional effects that are not reflected in conventional nutrient scoring systems (Drewnowski and Fulgoni, 2008; Weaver, 2014). Therefore, ingredient substitution can influence both the magnitude and the type of predicted nutrition and bioactivity outcomes, even before processing is considered.

Supplier variability poses a practical challenge for formulation design. Because no ingredients showed uniformly low variability, food product development teams should use lot-specific nutrient profiles rather than average composition values whenever possible. Although our database does not include bioactive compound data for commercial ingredients because such data are not publicly available, similar variability is likely. Another source of variability not considered in this study is lot-to-lot variability within the same supplier and product, driven by differences in seed genetics, growing conditions, harvest, and post-harvest handling.

The opposite responses of NRF_9_._3_ and FRAP to extrusion reflect two well-characterized mechanisms: thermal degradation of heat-labile vitamins (Camire et al., 1990; Riaz et al., 2009; Coelho, 2002) and mechanical release of bound phenolics from disrupted cell walls (Nayak et al., 2015; Song et al., 2022; Zeng et al., 2016) (Fig. 2a). The greater NRF_9_._3_ sensitivity of fruit- and vegetable-enriched formulations is consistent with their higher starting concentrations of vitamins A, C, and E, which are the nutrients most vulnerable to first-order thermal degradation.

Because ingredient sourcing dominated NRF_9_._3_ variability (typically 3- to 11-fold more important than processing; Fig. 2b), raw material specifications and sourcing control are as important as process design for achieving consistent nutritional outcomes. At the same time, some formulations exhibited substantial processing-driven changes, suggesting that process optimization can be a dominant lever when thermal sensitivity is high.

The uncertainty propagation analysis provides actionable guidance beyond the aggregate finding that sourcing dominates processing. The nutrient-level decomposition (Fig. 3c) shows that vitamin A, calcium, and iron are the primary drivers of NRF_9_._3_ instability, while protein and vitamin C contribute little uncertainty. This suggests that targeted supplier specifications for a small number of nutrients, rather than blanket quality control across all analytes, could substantially reduce nutritional variability in finished products. Furthermore, the formulation-dependent nature of the sourcing-to-processing ratio implies that the optimal quality control strategy differs by product type: starch-dominant formulations benefit most from tighter raw material specifications, whereas vitamin-rich formulations benefit more from process optimization.

The clustering of optimal conditions into mild versus intensive strategies reflects the trade-off between phenolic release (favored by higher severity) and vitamin retention (favored by lower severity), the balance point of which shifts with each formulation's starting composition. Formulations rich in heat-labile vitamins (A, C, E) generally favored mild conditions (≤113 °C, ≤35 s) to minimize degradation, whereas formulations rich in bound phenolics from whole grains such as barley and sorghum benefited from more intensive processing (>130 °C) to maximize antioxidant release through cell-wall disruption. The kinetic models (Fig. 4b) reveal a processing severity region where free phenolic release is maximized before vitamin losses accelerate, and this region shifts depending on each formulation's initial phenolic reservoir and vitamin profile. Formulations with inherently robust compositions showed negligible benefit from optimization, suggesting that reformulation or tighter raw material specifications may be more effective levers for these systems.

It should also be noted that this study presents a computational framework, and its predictions require future experimental validation. Extrusion in this study was parameterized by temperature and residence time only; incorporating moisture content, specific mechanical energy, screw configuration, and shear rate would improve model fidelity for industrial implementation. Also, the present work was focused on nutritional and chemical outcomes. Physical and sensory quality attributes, such as expansion, bulk density, and texture, are examined in a separate dedicated study. In practical applications, the two models can be integrated to identify formulations and extrusion conditions that improve nutritional and health performance while maintaining acceptable sensory quality.

## 5 Conclusion

This study presents a computational framework that integrates an ingredient-level compositional database, mechanistic models, and machine learning to optimize nutrition and bioactivity in extruded foods. Across the 20 selected formulations, modeling consistently predicted decreased NRF_9.3_-based nutritional quality and increased antioxidant capacity. Monte Carlo uncertainty propagation revealed that ingredient sourcing variability typically dominated processing-induced changes in NRF_9_._3_, though vitamin-rich formulations were exceptions where process control mattered most. Formulation-specific optimization of extrusion temperature and residence time was predicted to improve the combined nutrition–antioxidant score by up to 44%, with the largest gains in bioactive-rich systems. The results suggest that ingredient sourcing control and process optimization are complementary strategies whose relative importance depends on formulation composition. Experimental validation and integration of sensory prediction models will be important next steps.

## Author contributions

X.Z.: Writing – original draft, Methodology, Formal analysis, Data curation. K.N.: Writing – review & editing, Methodology, Formal analysis, Data curation. P.G.: Writing – review & editing, Methodology, Formal analysis. I.T.: Writing – review & editing, Supervision, Formal analysis, Methodology, Project administration, Funding acquisition, Conceptualization.

## Data availability

The ingredient composition database (Supplementary Table S2), formulation table (Supplementary Table S1), and the Bioactivity Food Link (BFL) dataset used in this study are provided as Supplementary Information.

## Code availability

The code for data extraction, machine learning, and optimization is available at https://github.com/IBPA/Food-Processing-Extrusion-Modeling.

## Declaration of competing interest

The authors declare that they have no known competing financial interests or personal relationships that could have appeared to influence the work reported in this paper.
