# Supplementary material for: Computational optimization of food formulation and extrusion conditions for nutritional quality and antioxidant capacity
Source: Curr Res Food Sci. 2026 Jul 22;13:101509. doi: 10.1016/j.crfs.2026.101509 (PMC13452384; doi:10.1016/j.crfs.2026.101509)
Supplement: Supplementary file 1 [file mmc1.docx]

**Computational optimization of food formulation and extrusion conditions for nutrient density and antioxidant capacity**

Xu Zhou^1,2#^, Keer Ni^1,2#^, Pranav Gupta^1,2^ & Ilias Tagkopoulos^1,2*^

^1^Department of Computer Science & Genome Center, University of California, Davis, 95616, USA

^2^USDA/NSF AI Institute for Next Generation Food Systems (AIFS), Davis, 95616, USA

**SUPPLEMENTARY INFORMATION**

Contents

[1. SUPPLEMENTARY TEXT	2](#_Toc224935140)

[2.1. Kinetic model	2](#_Toc224935141)

[2.2. Random forest	3](#_Toc224935142)

[2.2.1 Dataset curation and target variable specification	3](#_Toc224935143)

[2.2.2 Bioactivity Food Link (BFL): potency and structural representation	4](#_Toc224935144)

[2.2.3 Baseline heuristics and proxy scoring	4](#_Toc224935145)

[2.2.4 Feature engineering for the Bioactivity Prediction Model (BPM)	5](#_Toc224935146)

[2.2.5 Model training and evaluation	6](#_Toc224935147)

[REFERENCES	7](#_Toc224935148)

[2. SUPPLEMENTARY FIGURES	8](#_Toc224935149)

# 1. SUPPLEMENTARY TEXT

## 2.1. Formulation generation

Candidate dry-mix formulations were generated with a large language model (OpenAI GPT-5.2) using the structured prompt below, which encodes the 28-ingredient set grouped by class, the sum-to-one constraint, and conservative per-class bounds for lipid- and fiber-rich ingredients. Diversity was defined as coverage across ingredient classes and composition ranges, operationalized as seven formulation archetypes. After generation, candidate formulations were screened programmatically for the sum-to-one and per-class bounds; redundant recipes were removed as near-duplicate compositions (pairwise cosine similarity above 0.95 on the ingredient-fraction vectors); and the remaining set was manually curated for archetype coverage, yielding the 20 formulations in Table S1.

**Formulation-generation prompt:**

```
[SYSTEM]
You are a food-formulation assistant for extruded breakfast cereals. You design dry-mix
formulations (mass fractions of a fixed ingredient set) that are feasible for twin-screw
extrusion and nutritionally diverse. Obey all constraints exactly and return only valid JSON.

[USER]
Task: Propose 20 diverse dry-mix breakfast-cereal formulations using only the 28 ingredients
below. Each formulation is a set of mass fractions that sum to exactly 1.000.

Ingredient set (by class):
- Cereal/grain flours: Corn flour, Wheat flour, Rice flour, Oat flour, Barley flour,
  Rye flour, Millet flour, Buckwheat flour, Sorghum flour, Brown rice flour
- Starch: Tapioca starch
- Legume/plant proteins and flours: Pea protein, Rice protein, Chickpea, Lentil,
  Mung bean, Pea flour, Peanut flour, Almond flour
- Fiber: Pea fiber
- Fruit/vegetable powders: Tomato powder, Garlic powder, Onion powder, Strawberry powder,
  Apple powder
- Lipids/oils: Canola oil, Sunflower oil, Palm oil

Feasibility constraints (hard):
1. Mass fractions sum to 1.000 (tolerance 0.001).
2. Each formulation uses 2 to 6 ingredients (nonzero); all others 0.
3. A cereal/grain flour or starch is the base: combined cereal/grain + starch >= 0.55.
4. Tapioca starch, if used: 0.05 to 0.20.
5. Single protein/legume ingredient <= 0.40; total protein/legume class <= 0.50.
6. Pea fiber <= 0.10.
7. Each fruit/vegetable powder <= 0.15; total fruit/vegetable class <= 0.20.
8. Each oil <= 0.05; total oil class <= 0.05.
9. Nonzero fractions >= 0.05, rounded to 0.05.

Diversity requirements:
- Span archetypes: starch-dominant, multigrain, protein-fortified, fiber-fortified,
  fruit-enriched, savory-vegetable, whole-grain-antioxidant.
- Vary the dominant grain across recipes (reuse any base grain in at most ~5 recipes).
- Cover a broad range of total protein and total fruit/vegetable content.

Output (JSON only):
[
  {"name":"<short name>",
   "archetype":"<one of the seven archetypes>",
   "composition":{"<ingredient>":<fraction>, ...}},
  ... 20 items ...
]
Include only nonzero ingredients; fractions sum to 1.000; use the exact ingredient names;
return no text outside the JSON array.
```

## 2.2. Kinetic model

The coupled mass balances are:

| $\frac{\text{d}\text{C}_{\text{b}}}{\text{dt}}\text{=-}\text{k}_{\text{b}}\left( \text{T} \right)$ | S1 |
| --- | --- |
| $\frac{\text{d}\text{C}_{\text{f}}}{\text{dt}}\text{=}\text{k}_{\text{b}}\left( \text{T} \right)\text{-}\text{k}_{\text{f}}\left( \text{T} \right)\text{ }\text{C}_{\text{f}}$ | S2 |

With

| $\text{C}_{\text{b}}\left( \text{0} \right)\text{=}\text{C}_{\text{b,0}}\text{, }\text{C}_{\text{f}}\left( \text{0} \right)\text{=}\text{C}_{\text{f,0}}$ | S3 |
| --- | --- |

Both rate constants follow Arrhenius kinetics:

| $\text{k}_{\text{i}}\left( \text{T} \right)\text{=}\text{A}_{\text{i}}\text{exp​}\left( \text{-}\frac{\text{E}_{\text{a,i}}}{\text{RT}} \right)\text{, i}\text{∈}\text{\{b,f\}}$ | S4 |
| --- | --- |

Integrating Eq. (S1) gives:

| $\text{C}_{\text{b}}\left( \text{t} \right)\text{=}\text{C}_{\text{b,0}}\text{-}\text{k}_{\text{b}}\left( \text{T} \right)\text{ t}$ | S5 |
| --- | --- |

Because $\text{C}_{\text{b}}\text{≥0}$, we enforce the finite-reservoir constraint:

| $\text{C}_{\text{b}}\left( \text{t} \right)\text{=max​}\left( \text{C}_{\text{b,0}}\text{-}\text{k}_{\text{b}}\left( \text{T} \right)\text{ t, 0} \right)$ | S6 |
| --- | --- |

The time at which the bound pool is exhausted is:

| $\text{t}^{\text{*}}\text{=}\frac{\text{C}_{\text{b,0}}}{\text{k}_{\text{b}}\left( \text{T} \right)}$ | S7 |
| --- | --- |

Release operates only for $\text{t≤}\text{t}^{\text{*}}$. For $\text{t>}\text{t}^{\text{*}}$, the source term $\text{k}_{\text{b}}\left( \text{T} \right)$ in Eq. (S2) is set to zero.

Solve Eq. (S2) with constant coefficients using an integrating factor:

| $\frac{\text{d}\text{C}_{\text{f}}}{\text{dt}}\text{+}\text{k}_{\text{f}}\text{C}_{\text{f}}\text{=}\text{k}_{\text{b}}$ | S8 |
| --- | --- |

Integrating factor: $\text{μ}\left( \text{t} \right)\text{=}\text{e}^{\text{k}_{\text{f}}\text{t}}$. Then:

| $\frac{\text{d}}{\text{dt}}\left( \text{C}_{\text{f}}\text{e}^{\text{k}_{\text{f}}\text{t}} \right)\text{=}\text{k}_{\text{b}}\text{e}^{\text{k}_{\text{f}}\text{t}}$ | S9 |
| --- | --- |

Integrate from 0 to $\text{t}$, apply $\text{C}_{\text{f}}\left( \text{0} \right)\text{=}\text{C}_{\text{f,0}}$:

| $\text{C}_{\text{f}}\left( \text{t} \right)\text{=}\left( \text{C}_{\text{f,0}}\text{-}\frac{\text{k}_{\text{b}}}{\text{k}_{\text{f}}} \right)\text{e}^{\text{-}\text{k}_{\text{f}}\text{t}}\text{+}\frac{\text{k}_{\text{b}}}{\text{k}_{\text{f}}}\text{ }\left( \text{0≤t≤}\text{t}^{\text{*}} \right)$ | S10 |
| --- | --- |

The free concentration at depletion is:

| $\text{C}_{\text{f}}\left( \text{t}^{\text{*}} \right)\text{=}\left( \text{C}_{\text{f,0}}\text{-}\frac{\text{k}_{\text{b}}}{\text{k}_{\text{f}}} \right)\text{e}^{\text{-}\text{k}_{\text{f}}\text{t}^{\text{*}}}\text{+}\frac{\text{k}_{\text{b}}}{\text{k}_{\text{f}}}$ | S11 |
| --- | --- |

After depletion, $\text{k}_{\text{b}}\text{=0}$ and:

| $\frac{\text{d}\text{C}_{\text{f}}}{\text{dt}}\text{=-}\text{k}_{\text{f}}\text{C}_{\text{f}}$ | S12 |
| --- | --- |

Thus:

| $\text{C}_{\text{f}}\left( \text{t} \right)\text{=}\text{C}_{\text{f}}\left( \text{t}^{\text{*}} \right)\text{ }\text{e}^{\text{-}\text{k}_{\text{f}}\left( \text{t-}\text{t}^{\text{*}} \right)}\text{ }\left( \text{t>}\text{t}^{\text{*}} \right)$ | S13 |
| --- | --- |

Define $\text{t}_{\text{1}}\text{=min}\left( \text{t}_{\text{R}}\text{,}\text{t}^{\text{*}} \right)$. The exit bound concentration is:

| $\text{C}_{\text{b,out}}\text{=max}\left( \text{C}_{\text{b,0}}\text{-}\text{k}_{\text{b}}\text{t}_{\text{R}}\text{,0} \right)$ | S14 |
| --- | --- |

For $\text{k}_{\text{f}}\text{>0}$, the exit free concentration can be written compactly as:

| $\text{C}_{\text{f,out}}\text{=}\left[ \left( \text{C}_{\text{f,0}}\text{-}\frac{\text{k}_{\text{b}}}{\text{k}_{\text{f}}} \right)\text{e}^{\text{-}\text{k}_{\text{f}}\text{t}_{\text{1}}}\text{+}\frac{\text{k}_{\text{b}}}{\text{k}_{\text{f}}} \right]\text{ }\text{e}^{\text{-}\text{k}_{\text{f}}\left( \text{t}_{\text{R}}\text{-}\text{t}_{\text{1}} \right)}$ | S15 |
| --- | --- |

Equivalent piecewise form:

| $\text{C}_{\text{f,out}}\text{=}\left\{ \begin{matrix} \left( \text{C}_{\text{f,0}}\text{-}\frac{\text{k}_{\text{b}}}{\text{k}_{\text{f}}} \right)\text{e}^{\text{-}\text{k}_{\text{f}}\text{t}_{\text{R}}}\text{+}\frac{\text{k}_{\text{b}}}{\text{k}_{\text{f}}}\text{,} & \text{t}_{\text{R}}\text{≤}\text{t}^{\text{*}} \\ \left[ \left( \text{C}_{\text{f,0}}\text{-}\frac{\text{k}_{\text{b}}}{\text{k}_{\text{f}}} \right)\text{e}^{\text{-}\text{k}_{\text{f}}\text{t}^{\text{*}}}\text{+}\frac{\text{k}_{\text{b}}}{\text{k}_{\text{f}}} \right]\text{e}^{\text{-}\text{k}_{\text{f}}\left( \text{t}_{\text{R}}\text{-}\text{t}^{\text{*}} \right)}\text{,} & \text{t}_{\text{R}}\text{>}\text{t}^{\text{*}} \end{matrix} \right.$ | S16 |
| --- | --- |

**Table S3.** Summary of Arrhenius kinetic parameters used in the mechanistic models.

| **Compound** | **Reaction** | **E_a_ (kJ/mol)** | **A** | **Order** |
| --- | --- | --- | --- | --- |
| Vitamin A | Degradation | 48.5 | 5.52 × 10^4^ | 1st |
| Vitamin C | Degradation | 42.2 | 1.69 × 10^4^ | 1st |
| Vitamin E | Degradation | 49.4 | 1.36 × 10^5^ | 1st |
| Ferulic acid | Release | 50.0 | 2.94 × 10^5^ | 0th |
| Ferulic acid | Degradation | 90.0 | 1.39 × 10^9^ | 1st |
| Protocatechuic acid | Release | 48.0 | 1.10 × 10^5^ | 0th |
| Protocatechuic acid | Degradation | 110.0 | 2.45 × 10^13^ | 1st |
| p-Hydroxybenzoic acid | Release | 58.9 | 2.29 × 10^6^ | 0th |
| p-Hydroxybenzoic acid | Degradation | 99.1 | 3.56 × 10^10^ | 1st |
| Vanillic acid | Release | 8.3 | 2.25 | 0th |
| Rutin | Release | 39.6 | 3.27 × 10^6^ | 0th |
| Rutin | Degradation | 47.5 | 1.64 × 10^6^ | 1st |

## 2.3. Bioactivity prediction model

This section describes the development of a supervised learning model, similar to that reported in [1] to predict formulation-level antioxidant capacity (FRAP) from processing-adjusted chemical composition data. The approach proceeded in three stages: (i) dataset assembly and target variable definition, (ii) evaluation of baseline heuristic approaches using compound-level potency data, and (iii) training of a Random Forest regressor on concentration-derived features. A conceptual overview of the prediction framework is shown in Figure S1.

### **2.3.1 Dataset curation and target variable specification**

Food- and ingredient-level antioxidant measurements (FRAP, ORAC, DPPH, ABTS) were compiled from FoodAtlas (https://www.foodatlas.ai/). The compiled dataset comprised 189 foods from FoodAtlas, along with additional extrusion-condition-specific food samples from three literature sources [2], [3], [4]. Although this sample size is modest for machine learning, it reflects the current scarcity of extrusion studies that report both detailed chemical composition and standardized antioxidant endpoints under controlled processing conditions. FRAP was selected as the primary supervised learning target because it provides a direct, electron-transfer-based measure of reducing power and had the most complete coverage across the sampled food matrices. Values were standardized to mmol Fe²⁺ equivalents per 100 g (dry basis) and log-transformed to reduce right-skew and stabilize variance for regression modeling. DPPH, ORAC, and ABTS measurements were retained only when reported in Trolox equivalent (TE) units to ensure commensurability across sources.

Although antioxidant values in the source studies were measured at the extract level, modeling was conducted at the ingredient composition level. Specifically, chemical concentrations for each ingredient were first adjusted for processing effects, then summed across all ingredients in a formulation to produce a single formulation-level feature vector. This representation enables prediction under extrusion conditions spanning 0-120 s and 100-200 °C.

### **2.3.2 Bioactivity Food Link (BFL): potency and structural representation**

Before developing a supervised model, a compound-level potency framework was constructed to test whether food-level antioxidant capacity could be approximated through direct aggregation of individual chemical activities. For each chemical in the composition tables, antioxidant-relevant potency data were retrieved from ChEMBL (https://www.ebi.ac.uk/chembl/), with supporting identifiers and structures obtained from PubChem when needed. Potency was represented as pChEMBL[5] values, defined as the negative logarithmic transformation of standardized half-maximal activity concentrations (e.g., IC₅₀, EC₅₀). Records were retained only when standardized activity types and compatible units were available.

Assays were filtered using curated keyword rules to ensure explicit antioxidant, redox, or free-radical relevance, including terms related to DPPH, ABTS/TEAC, ORAC, and hydroxyl radical scavenging. Non-redox pharmacological assays were excluded. SMILES strings were resolved via identifier mapping and converted to 2048-bit Morgan fingerprints (ECFP4, radius = 2) using RDKit (v. 2023.09). Fingerprints were treated as binary feature vectors without scaling.

### **2.3.3 Baseline heuristics and proxy scoring**

Two aggregation strategies were evaluated using the Bioactivity-Food Link (BFL) framework to test whether food-level FRAP could be approximated without supervised training.

The first strategy used heuristic summarization of potency-only measures, analogous to HAS-style scoring, without accounting for compound concentrations. The second strategy computed linear concentration-weighted proxy scores of the form:

*S_assay = Σ [Aᵢ] × 10^(pChEMBLᵢ)*	(S17)

where Aᵢ is the concentration of chemical i in the food and 10^(pChEMBLᵢ) maps the logarithmic potency back to linear scale. Both formulations assume additive, independent contributions from each compound.

Neither approach predicted food-level FRAP (R² ≈ 0). The failure likely reflects incomplete concentration coverage in public databases, non-additive interactions among antioxidant compounds (e.g., synergism or antagonism), variability in potency measurements across laboratories and assay conditions, and nonlinear effects of thermal processing on compound availability. These findings motivated a supervised learning approach trained directly on processing-adjusted concentration features rather than externally derived potency values.

### **2.3.4 Feature engineering for the Bioactivity Prediction Model (BPM)**

In contrast to the baseline proxies, the Bioactivity Prediction Model (BPM) was trained using processing-adjusted chemical concentrations as the sole input features. No potency descriptors or structural fingerprints from the BFL framework were included.

Chemical concentrations were first adjusted using the mechanistic kinetic models described in Section 2.1 to account for thermal degradation and phenolic release during extrusion. Adjusted values were then aggregated within each ingredient and summed across all ingredients in a given formulation to construct a fixed-length formulation-level feature vector. Each element of the resulting vector represents the estimated post-extrusion concentration of a single chemical compound in the complete formulation. This representation preserves relative dose contributions under defined extrusion conditions while maintaining consistent dimensionality across all samples.

Feature refinement was then performed using mean decrease in impurity (Gini importance) scores averaged across all trees in the ensemble. Variables with importance scores consistently below the median importance were removed in a single pass. After refinement, the final formulation-level feature space comprised 18 variables, reduced from an initial pool of 62 candidates.

### **2.3.5 Model training and evaluation**

The BPM was implemented as a Random Forest regressor, an ensemble method that constructs multiple decision trees on bootstrap-drawn subsamples of the training data and averages their predictions to reduce overfitting and improve generalization. Random Forest was selected for this application because of its robustness to small sample sizes, tolerance of correlated features, and ability to provide interpretable feature importance rankings.

Model stability was assessed using 100 iterations of repeated random 80/20 train–test splits. Splitting was performed at the formulation level, ensuring that all extrusion conditions associated with a given formulation appeared exclusively in either the training or the test set to prevent information leakage.

Hyperparameters were optimized within each training split using grid search with 3-fold cross-validation (GridSearchCV, scikit-learn v. 1.3). The search grid comprised: number of trees (100, 200, 300), maximum depth (None, 10, 20, 30), minimum samples for splitting (2, 5, 10), minimum samples per leaf (1, 2, 4), and bootstrap sampling (True, False), yielding 216 candidate configurations per split. Given the modest training set size (~24 samples per split), inner cross-validation folds contained approximately 16 samples each. To mitigate the risk of overfitting the hyperparameter search, performance stability was verified across the 100 outer splits. The configuration selected most frequently used 200 trees, unrestricted depth (max_depth = None), min_samples_split = 10, min_samples_leaf = 1, and bootstrap = True. All preprocessing and feature selection steps were conducted within each training split to prevent data leakage.

Across the 100 held-out test sets, the model achieved a mean Pearson correlation coefficient (PCC) of 0.87 ± 0.06, mean RMSE of 0.42 ± 0.09, and mean R² of 0.75 ± 0.08 (Figure S2). Residual analysis indicated no systematic trend with predicted value, though slightly increased variance was observed at higher FRAP values.

To contextualize the choice of Random Forest, nine additional regression algorithms were evaluated under identical preprocessing and split-validation protocols: Gradient Boosting, AdaBoost, XGBoost, Support Vector Regression (RBF kernel), LightGBM, ElasticNet, Lasso, Ridge, and multilayer perceptrons (shallow and deep architectures). Performance was assessed across six metrics: test R², RMSE, MAE, Pearson correlation, Spearman correlation, and overfitting resistance (defined as the difference between training and test R²). All models used the same grid-search-based hyperparameter tuning protocol. Given the modest sample size, this comparison primarily serves to confirm that ensemble tree-based methods are well-suited to the dataset structure rather than to establish definitive rankings among all algorithms. As shown in Figure S3, Random Forest achieved the highest normalized score across all six metrics, with particularly strong performance in rank-order preservation (Spearman correlation) and resistance to overfitting. Neural network architectures (shallow and deep MLP) performed poorly, consistent with their known sensitivity to limited training data.

**Extrusion-specific evaluation.** Because the intended application of the model is the prediction of antioxidant capacity under extrusion, we additionally evaluated performance on the subset of 30 extrusion-specific samples (heat-treated flours measured at defined temperature–time conditions). Using a held-out, leave-one-study-out scheme, the model explained approximately 46% of the variance on this subset (R^2^ = 0.46), lower than the full-dataset estimate (R^2^ = 0.75) and reflecting the small number of extrusion-specific samples and their narrow compositional range. We therefore interpret the model as a screening-level predictor that captures the direction and relative ranking of extrusion-induced antioxidant changes rather than their exact magnitude (Table S4).

**Table S4.** FRAP model performance on the full BFL dataset and on the extrusion-specific subset.

| **Evaluation set** | **Samples (n)** | **R^2^** |
| --- | --- | --- |
| Full BFL dataset (100× repeated 80/20 split-validation) | 275 | 0.75 |
| Extrusion-specific subset (leave-one-study-out) | 30 | 0.46 |

# REFERENCES

[1]	F. Li *et al.*, “A unified knowledge graph linking foodomics to chemical-disease networks and flavor profiles,” *Npj Sci. Food*, Jan. 2026, doi: 10.1038/s41538-025-00680-9.

[2]	D. Rico, A. B. Cano, and A. B. Martín-Diana, “Pulse-Cereal Blend Extrusion for Improving the Antioxidant Properties of a Gluten-Free Flour,” *Molecules*, vol. 26, no. 18, p. 5578, Sep. 2021, doi: 10.3390/molecules26185578.

[3]	L. Yang *et al.*, “Effect of Extrusion on the Hydrophilic Antioxidant Capacity of Four Whole Grains,” *J. Food Nutr. Res.*, vol. 2, no. 2, pp. 80–87, Mar. 2014, doi: 10.12691/jfnr-2-2-4.

[4]	M. H. Carlsen *et al.*, “The total antioxidant content of more than 3100 foods, beverages, spices, herbs and supplements used worldwide,” *Nutr. J.*, vol. 9, no. 1, p. 3, Dec. 2010, doi: 10.1186/1475-2891-9-3.

[5]	B. Zdrazil *et al.*, “The ChEMBL Database in 2023: a drug discovery platform spanning multiple bioactivity data types and time periods,” *Nucleic Acids Res.*, vol. 52, no. D1, pp. D1180–D1192, Jan. 2024, doi: 10.1093/nar/gkad1004.

# 2. SUPPLEMENTARY FIGURES

**Figure S1.** Conceptual overview of the bioactivity prediction framework. Chemical compounds within a food are linked to individual bioactivity measurements (y₁ … yₙ) and aggregated through a bioactivity-specific model to estimate food-level bioactivity.

**Figure S2.** Predicted vs. actual log-transformed FRAP values across held-out test sets. Each point represents a unique formulation–extrusion condition pair; error bars denote ± 1 SD across 100 repeated random splits. The dashed red line indicates perfect agreement (1:1 line). R² = 0.75, PCC = 0.87.

**Figure S3.** Normalized performance comparison across 10 regression algorithms evaluated under identical split-validation and hyperparameter tuning protocols. Scores were min–max normalized within each metric; green indicates better performance and red indicates worse. Random Forest achieved the highest aggregate score across all metrics.

**Figure S4**. Sensitivity of Monte Carlo uncertainty results to the assumed default coefficient of variation (CV) for ingredient–nutrient pairs lacking supplier-level standard deviations. Six CV levels (5%, 10%, 20%, 30%, 36%, 40%) were tested, each with n = 5,000 draws per formulation. (a) Sourcing-to-processing ratio for each formulation across CV levels. Dashed lines indicate equal contribution (ratio = 1) and the 5× threshold. (b) Number of formulations classified as sourcing-dominated (ratio ≥ 5), mixed (1–5), or processing-dominated (< 1) at each CV level, with median ratio shown on the secondary axis. The eight sourcing-dominated formulations remain unchanged across all CV levels; only the boundary between mixed and processing-dominated categories shifts modestly at higher CVs.

**Figure S5**. **Supplier-level variability in nutrient composition across 28 extrusion-relevant ingredients**. Boxplots show the distribution of reported nutrient values (per 100 g) for each ingredient across multiple supplier entries (sample size shown in parentheses). Nutrients include protein, calcium, energy, total dietary fiber, iron, magnesium, potassium, saturated fatty acids, sodium, vitamin C, and vitamin E (one nutrient per panel; pages 1–11). Boxes represent the interquartile range (IQR), center lines indicate medians, whiskers denote 1.5×IQR, and points indicate outliers. Substantial within-ingredient variability is observed for several nutrients, particularly minerals and vitamins, highlighting supplier-driven uncertainty in formulation composition

**Figure S6**. NRF with 95% Confidence Intervals from Ingredient Sourcing Uncertainty. Monte Carlo simulation of 5,000 runs.

**Figure S7**. Distribution of NRF for different formulation.
